# Peripheral Blood Lymphocyte Subpopulations in Patients Following Small Diameter Metal-On-Metal Total Hip Replacement at Long-Term Follow-Up

**DOI:** 10.3390/jcm9092877

**Published:** 2020-09-06

**Authors:** Tobias Reiner, Hester Haubenreisser, Elena Tripel, Nils Rosshirt, Reza Sorbi, Timo Albert Nees, Tobias Gotterbarm, Christian Merle, Babak Moradi, Sébastien Hagmann

**Affiliations:** 1 Center for Orthopedics, Trauma Surgery and Spinal Cord Injury, Heidelberg University Hospital, Schlierbacher Landstraße 200a, 69118 Heidelberg, Germany; hesterhaubenreisser@hotmail.com (H.H.); elena.tripel@med.uni-heidelberg.de (E.T.); nils.rosshirt@med.uni-heidelberg.de (N.R.); reza.sorbi@med.uni-heidelberg.de (R.S.); timo.nees@med.uni-heidelberg.de (T.A.N.); Christian.Merle@med.uni-heidelberg.de (C.M.); babak.moradi@med.uni-heidelberg.de (B.M.); 2 Department for Orthopaedics and Traumatology, Kepler University Hospital GmbH, Johannes Kepler University Linz, 4020 Linz, Austria; Tobias.Gotterbarm@kepleruniklinikum.at

**Keywords:** white blood cells, lymphocytes, blood metal ion levels, cobalt, chromium, titanium, Metasul, small head metal-on-metal THA, adverse local tissue reaction, ALTR

## Abstract

(1) Background: The objective of the present study was to investigate peripheral blood lymphocyte subpopulations in patients with small diameter metal-on-metal total hip arthroplasty (MoM THA) and elevated blood metal ion concentrations at long-term follow-up. The hypothesis was that increased blood metal ion levels or the presence of adverse local tissue reactions (ALTR) would be associated with changes in the peripheral expression of lymphocyte subpopulations, which could potentially serve as early diagnostic markers for metal wear related complications. (2) Methods: Peripheral blood samples were analyzed for leucocyte subgroups (CD3^+^, CD4^+^, CD8^+^, CD14^+^, CD16^+^/CD56^+^, CD25^+^/CD127^−^, CD19^+^, IFN-γ^+^, IL-4^+^ and IL-17A^+^ cells) in 34 patients with elevated blood metal ion levels (combined cobalt and chromium levels >2 µg/L) following small head MoM THA at a mean follow-up of 15.6 years. Fifteen patients with small head MoM THA and blood metal ion levels within the normal range and 15 patients with conventional ceramic-on-polyethylene THA served as control groups. In addition, blood metal ion levels and leucocyte subpopulations were compared between patients with and without adverse local tissue reactions (ALTR), which was investigated by MRI in 27 patients of the study cohort. (3) Results: There was a significant decrease in the levels of IFN-γ^+^ Type-1 T helper cells (Th1) in patients with MoM THA compared to the ceramic-on-polyethylene control group (*p* < 0.001). No statistically significant differences in the cell counts of other lymphocyte subpopulations were found between the three groups. Cobalt ion levels were significantly higher in patients with ALTR (*p* < 0.001) compared to the non-ALTR group, but no differences in the levels of lymphocyte subsets were found between the two groups. (4) Conclusions: No adverse systemic effects with respect to peripheral blood leucocyte subpopulations could be detected in the present study in patients following THA with a small diameter MoM articulation at long-term follow-up. We found a significant decrease of IFN-γ^+^ Th1 cells in patients with MoM THA compared to the control group, but no differences in the peripheral expression of leucocyte subpopulations were seen between patients with and without ALTR. Future studies with larger patient cohorts and additional histopathological investigations could help to better understand the role of Th1 cells and other cell lines of the adaptive immune system in the development of metal wear related complications after total joint replacement.

## 1. Introduction

Second generation metal-on-metal (MoM) bearings were reintroduced in the early 1990s in total hip arthroplasty (THA) in order to overcome the problems associated with polyethylene wear. Initial simulator studies demonstrated low volumetric wear rates, which made this bearing type popular especially in the treatment of young and active patients undergoing total hip replacement. At the end of the 2000s, MoM articulations were used in approximately 35% of all hip replacements in the United States [1]. However, high revision rates and the incidence of adverse local tissue reactions (ALTR) associated with metal wear led to a swift decrease in the use of MoM bearings in the subsequent years.

ALTR (also referred to as ARMD for adverse reaction to metal debris or pseudotumor) are closely linked with a cell-mediated immunological reactivity to metal wear particles and metal ions. Metal ions are generated by corrosion at the exposed metal surfaces and by corrosive degradation of metal wear particles. In-vivo, a passive oxide film is spontaneously formed on the metal surface that protects the implant from corrosion and inhibits the release of metal ions [2]. Due to repetitive mechanical stresses, for example at the modular taper junctions of total hip prosthesis, this protective oxide film gets disrupted which facilitates corrosion damage and metal ion release [3]. In a recently published investigation, Balachandran et al. described the folding mechanisms that take place during fretting at the taper junction between a co–chromium head and the femoral titanium taper. These folding mechanisms create raised shelves on the titanium surface that micro-plow the oxide film of the cobalt alloy and thereby promote tribocorrosion and metal ion release [4]. Accumulating metal ions are able to influence both bone metabolism and the immune system through different pathways, thereby contributing to the pathomechanisms of implant loosening and ALTR formation. It has been shown that metal ions are able to induce bone resorption by recruitment and activation of osteoclast precursor cells and that they can impede bone formation through direct inhibition of osteoblasts [5,6,7]. The immunological reactions to metal wear products can be generally described as an immune response driven either by the innate (non-specific) or the adaptive (specific) immune system [8]. A typical cellular driven immune response of the innate immune system to metal wear particles is dominated by an infiltration of macrophages and giant cells, which can cause a particle induced granulomatous tissue reaction. The inflammatory reaction to metal wear products driven by the adaptive immune system is controlled by CD3^+^ T lymphocytes, in particular CD4^+^ T helper cells and CD8^+^ cytotoxic T cells [8,9]. The histopathological patterns of these ALTR are characterized by a chronic inflammation with a marked lymphocytic infiltrate, which is typically dominated by B and T lymphocytes with a perivascular and diffuse distribution. This characteristic histopathological appearance determined the term ALVAL (aseptic lymphocyte-dominated vasculitis-associated lesions), which is also commonly used to describe these metal wear related tissue reactions [8,10].

Metal ions are able to pass into the blood circulation, where they can bind to serum proteins and form hapten-like complexes. These metal–protein-complexes can be recognized by T-lymphocytes as an antigens, which might trigger a specific immune response [11,12,13,14,15]. Some previously published studies have investigated the relationship between circulating metal ions and lymphocyte counts in patients with MoM THA. Catelas et al. found lower percentages of memory T cells in the peripheral blood of patients with failed MoM hip replacement, suggesting a type IV hypersensitivity reaction to be associated with the development of ALTR [16]. A study by Penny et al. noticed a depressive effect of increased systemic cobalt ion concentrations on circulating T lymphocytes in a randomized controlled trial after two years [17]. Other authors demonstrated a reduction in CD8^+^ cytotoxic T cells in patients with elevated cobalt and chromium ion concentrations at short-term follow-up [18]. However, little is known about the effects of a small head metal-on-metal bearing on peripheral lymphocyte subpopulations in patients with a well-functioning total hip replacement at long-term follow-up.

Therefore, the purpose of the present study was to investigate the peripheral expression of lymphocyte subpopulations in the blood of patients with a small head MoM THA and elevated blood metal ion concentrations at a minimum follow-up of 10 years. Patients with small head MoM THA who demonstrated metal ion levels within the normal range and patients with a conventional ceramic-on-polyethylene (CoP) THA served as control groups. Our hypothesis was that increased blood metal ion levels would be associated with changes in the levels of peripheral lymphocyte subpopulations in accordance to previous studies that have reported a suppressive effect of metal ions on peripheral T lymphocytes [16,17,18,19,20]. The second aim of the study was to compare levels of lymphocyte subpopulations between patients with and without ALTR at long-term follow-up. Our hypothesis was that the presence of a suspected implant-related T cell-mediated hypersensitivity reaction in patients with ALTR would be associated with changes in the levels of systemic lymphocyte subsets.

## 2. Methods

### 2.1. Study Design and Patient Groups

The study was performed according to local ethics board approval and written informed consent for each patient was obtained prior to study enrolment. Patients were recruited from a consecutive cohort of 301 patients, who underwent total hip arthroplasty with a 28-mm Metasul metal-on-metal bearing (Metasul^®^, Zimmer Biomet, Warsaw, IN, USA) between April 1995 and November 2001 at our institution. The patient collective was previously evaluated with regard to long-term survivorship and metal ion exposure as part of a previously published study [21]. Blood metal ion levels were available in 191 of these patients at a mean follow-up of 14 years and 50% of patients demonstrated combined blood metal ion levels of cobalt and chromium >2 µg/L.

The inclusion criteria for the present study were (1) uni- or bilateral cementless THA with a 28-mm Metasul metal-on-metal bearing; (2) a minimum follow-up of 10 years; and (3) increased blood metal ion levels, which were defined as a combined cobalt and chromium ion concentration >2 µg/L. Exclusion criteria included patients following contralateral hip resurfacing or large diameter metal-on-metal THA; additional metal implants to avoid other sources of cobalt or chromium metal ion release; patients with history of occupational exposure to cobalt or chromium; a known metal allergy; previously diagnosed blood dyscrasias or malignancies; diagnosis of any inflammatory autoimmune disease including rheumatoid arthritis; intake of DMARDs; HIV infection; pregnancy; and suspected joint infection. A total of 34 patients met the inclusion/exclusion criteria and agreed to participate in the present study. Fifteen patients with well-functioning small head MoM THA and blood metal ion levels within the normal range (defined as combined cobalt and chromium ion concentrations <1 µg/L) and 15 patients with conventional THA with a ceramic-on-polyethylene bearing who did not meet any of the exclusion criteria served as control groups.

A 28-mm MoM articulation (Metasul^®^, Zimmer Biomet, Warsaw, IN, USA) was used in this cohort, which consists of a forged, high-carbide (0.2–0.25%) co–chromium alloy liner, embedded in a polyethylene insert, in combination with a 28-mm co–chromium alloy femoral head. The general indication for the use of a Metasul bearing at that time was young patient age with a high expected physical activity level. The Metasul articulation was used in combination with a cementless press-fit titanium acetabular component and a cementless straight tapered femoral titanium stem with a standard 12/14 mm Euro taper in all hips. The ceramic-on-polyethylene bearing in the control group consists of a highly crosslinked polyethylene insert (Durasul^®^, Zimmer Biomet, Warsaw, IN, USA) which was used in combination with a 32-mm ceramic femoral head (Biolox^®^ forte, CeramTec GmbH, Plochingen, Germany) and a cementless titanium alloy femoral stem.

### 2.2. Clinical and Radiological Follow-Up

The demographic data for each patient were documented at the time of follow-up. In the metal-on-metal group, clinical assessment was performed using the modified Harris Hip Score (HHS). Standard pelvis anteroposterior and lateral radiographs of the hip were evaluated to rule out implant loosening. To investigate differences in frequencies of lymphocyte subpopulation in patients with and without adverse local tissue reactions (ALTR), we retrospectively evaluated metal artifact reduction sequence magnetic resonance imaging (MARS MRI) regarding the presence of ALTR. MARS MRI was available in 27 patients of the study group (79%). MRI was performed on a 3T whole body scanner (MAGNETOM Verio; Siemens Healthcare; Germany) with dedicated phased array coils and metal artifact reducing sequences (syngo WARP) in all patients, as part of a previously published study [22]. Any cystic or solid mass with relation to the hip joint was defined as ALTR according to the MRI classification system of Hauptfleisch et al. [23]. However, isolated fluid distensions of the trochanteric bursa and small fluid collection at the femoral neck without bulging and without nodular appearance of the joint capsule were not included [22].

### 2.3. Blood Metal Ion Analysis

Blood samples were collected at time of follow-up using specific needles and vacuum tubes suitable for metal ion analysis (Sarstedt AG & Co. KG, Nuembrecht, Germany; Refs. 58.1162.600 and 01.1604.400). The first 5 mL of blood were discarded in order to avoid contamination and blood samples were stored at −20 °C before analysis. Whole blood metal ion analysis was performed using high-resolution inductively coupled plasma-mass spectrometry (HR-ICP-MS, Element 2, Thermo Fisher Scientific, Bremen, Germany) at the Geochemical Laboratories at Heidelberg University. Metal ion analysis was repeated three times and mean values of metal ion concentrations were calculated for each sample. Detection limits of 0.005 µg/L for cobalt, 0.02 µg/L for chromium and 0.06 µg/L for titanium were previously established for this method [24].

### 2.4. Sample Preparation and Flow Cytometry Analysis

Blood samples were collected at the time of follow-up through puncture of a peripheral vein under sterile conditions into two 9-mL polypropylene K-EDTA tubes (Monovette-S 9 mL EDTA-K; Sarstedt, Nuembrecht, Germany). Each blood sample was immediately processed to retain high cell viability. Peripheral blood mononuclear cells (PBMC) were isolated under aseptic conditions using Ficoll-Paque^TM^ PLUS (GE Healthcare, Chicago, Illinois, USA) density gradient centrifugation. Mononuclear cells were washed twice in magnetic-activated cell sorting (MACS) staining buffer, blocked with Fetal calf serum (FCS) blocking reagent to prevent unspecific binding and stained for 30 min at 4 °C with PE-Cy7 conjugated monoclonal antibodies (mAB) against CD3 (clone SK7, BD Biosciences, USA); APC-Cy7 conjugated mAb against CD4 (clone RPA-T4, BD Biosciences, USA); VioBlue-labeled mAb against CD8 (clone BW135/80, Miltenyi Biotec, Germany); FITC-labeled mAb against CD14 (clone M5E2, BD Biosciences, USA); PE-Cy7 labeled mAb against CD16 (clone 3G8, BD Biosciences, USA); PE-labeled mAb against CD19 (clone LT19, Miltenyi Biotec, Germany); APC labeled mAb against CD56 (clone AF12-7H3, Miltenyi Biotec, Germany); FITC-labeled mAb against CD4 (clone RPA-T4, BD Biosciences, Bergen, NJ, USA); PE-labeled mAb against CD25 (clone M-A251, BD Biosciences, USA); and PerCP-Cy5.5-labeled mAb against CD127 (clone RDR5, eBioscience, San Diego, CA, USA). The cells were washed again and taken into a final volume of 200 μL MACS staining buffer. Before flow cytometry detection, cells were stained with 7-aminoactinomycin D (7-AAD; eBioscience, San Diego, CA, USA) at a concentration of 0.5 μg/mL, to exclude cell debris and dead cells. Multi-color flow cytometry analysis was used to identify leucocyte subtypes according to their distinct cell surface marker expression (Table 1). Flow cytometry was performed using a MACS-Quant Analyzer (Miltenyi Biotec, Bergisch Gladbach, Germany). Data analysis was performed using FlowJo^TM^ version 10.6.1 (TreeStar, Inc., Ashland, OR, USA). Mononuclear cells (MNC) were gated based on their forward- and side-scatter profiles. MNC were defined by their surface marker expression as CD4^+^ T helper (Th) cells, CD4^+^ CD25^+/high^CD127^low/-^ regulatory T cells (Treg), CD8^+^ cytotoxic T (Tc) cells, CD14^+^ macrophages, CD19^+^ B cells and CD16^+^CD56^+^ natural killer (NK) cells.

For intracellular immunostaining, CD3^+^ MACS-isolated T cells were taken into culture at a final density of 10^6^ T cells/mL and incubated for 10 h at 37 °C/5% CO_2_. Cell cultures were stimulated with phorbol-myristate-acetate (PMA) (50 ng/mL) and ionomycin (1 μg/mL, BD Biosciences, USA). After 4 h of activation, brefeldin A (5 μg/mL, Sigma-Aldrich, Germany) was added for another 6 h to prevent secretion and allow intracellular detection of cytokines. After a total of 10 h of activation, T cells were collected, washed twice in FACS buffer and blocked with FCS blocking reagent. Surface marker staining was performed with PE-Cy7 conjugated mAb against CD3 (clone SK7, BD Biosciences, USA); APC-Cy7 conjugated mAb against CD4 (clone RPA-T4, BD Biosciences, USA); and VioBlue-labeled mAb against CD8 (clone BW135/80, Miltenyi Biotec, Germany) at 4 °C for 30 min. T cells were then fixed and permeabilized using cytofix/cytoperm reagent (BD Biosciences, USA), stained with APC-labeled anti-IFN-γ (clone B27; isotype control clone MOPC-21; BD Biosciences, USA); FITC-labeled anti–IL-4 (clone MP4-2502; isotype control clone R3-34; BD Biosciences, USA); and PE-labeled anti-IL-17A (clone N49-653; isotype control clone MOPC-21; BD Biosciences, USA) and measured by flow cytometry. CD3^+^CD4^+^CD8^−^ T cell subsets were defined by intracellular cytokine staining and surface marker expression, respectively, and described as: IFN-γ^+^ Type 1 (Th1) T helper cells, IL-4^+^ Type 2 T helper cells (Th2), IL-17A^+^ T helper cells (Th17) and CD25^+/high^CD127^low/-^ regulatory T cells (Treg). Cut-off was defined based on isotype controls, as previously described [25,26].

### 2.5. Statistical Analysis

Statistical analysis was performed using the software SPSS^®^ for Windows^®^ (version 22.0; SPSS IBM Corp., Chicago, IL, USA) and GraphPad Prism^®^ (version 6.0, GraphPad Software, San Diego, CA, USA). Data were evaluated descriptively as arithmetic mean, standard deviation, median, minimum and maximum. The Shapiro–Wilk test was used to assess if the data conformed to the assumption of normality. When data distributions were normal, statistical analysis was performed using the analysis of variance (ANOVA) and Tukey–Kramer post hoc tests for comparison of lymphocyte counts and demographic parameters between the three groups. When data distributions were not normal, the Kruskal–Wallis and two-sided Mann–Whitney U-Test were used. Bivariate analysis was performed using the independent t test for comparison of continuous variables and the Fisher’s test for comparison of categorical variables between the two groups. Correlation analysis between lymphocyte cell counts and blood metal ion concentrations was performed using Spearman correlation coefficient and multivariate linear regression analysis. Additionally, the relationship between the presence of ALTR and levels of lymphocyte subpopulations was assessed using logistic regression analysis. Correlation was defined as poor (0.00–0.20), fair (0.21–0.40), moderate (0.41–0.60), good (0.61–0.80) or excellent (0.81–1.00). All tests were two-sided and a *p*-value < 0.05 was considered significant.

## 3. Results

### 3.1. Description of the Study Cohort

In total, 64 patients (28 female and 36 male patients) with a mean patient age at time of follow-up of 67 years (range 27–82 years) were included in the present study. Demographic data of the study group and the two control groups are summarized in Table 2. Indication for THA was primary osteoarthritis in 45 patients (70%), post-traumatic osteoarthritis in 2 patients (3%), secondary osteoarthritis caused by congenital dysplasia in 14 patients (22%) and secondary osteoarthritis caused by avascular necrosis of the femoral head in 3 patients (5%). No statistically significant difference in mean patient age at time of follow-up and mean body mass index was found between the groups. The percentage of male patients was higher in the MoM control group as compared to the MoM study group and the CoP group, however the difference was not statistically significant. The follow-up duration was significantly shorter in the CoP group, but no significant difference in follow-up duration was found between the MoM study group and the MoM control group (*p* = 0.080). Combined cobalt and chromium ion levels differed significantly between the MoM study group and the control groups (*p* < 0.001) but were comparable between the control groups (*p* = 0.238).

### 3.2. Clinical and Radiological Evaluation

The mean Harris Hip Score of the cohort was 89 points (range 50–100) at time of follow-up. No statistically significant difference in mean HHS was found between the study and the control group and most of the patients were asymptomatic with regard to the investigated hip with a mean HHS of 90 points in the study group. No implant showed radiographic signs of loosening. MARS-MRI demonstrated pseudotumor formation in 11 of the 27 investigated hips (41%). ALTR consisted mainly of cystic lesions with a mean lesion size of 4.7 cm^3^ (range 0.3–18.3 cm^3^). They were classified as Type-1 lesions in six hips; as Type-II lesions in four hips; and as Type-III lesion in one hip according to the classification system of Hauptfleisch et al. [23]. Eight of the 11 patients with ALTR were asymptomatic regarding the investigated total hip prosthesis. No significant difference in mean HHS was found between patients with ALTR and patients without ALTR (*p* = 0.933).

### 3.3. Blood Metal Ion Analysis

**Figure 1:**
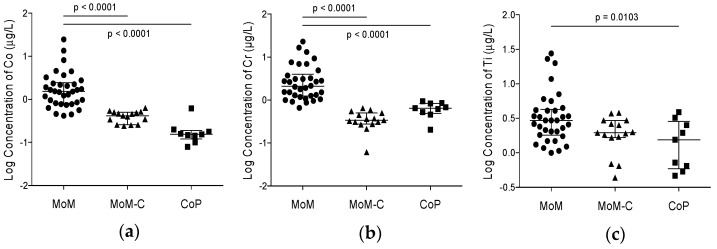
Vertical scatter plots showing whole blood metal ion concentrations after logarithmic transformation of the data for comparison between the three groups of: (**a**) cobalt; (**b**) chromium; and (**c**) titanium. Lines with *p* values connect groups with significant differences. Horizontal bars show medians with interquartile ranges. MoM, Metal-on-Metal study group; MoM-C, Metal-on-Metal control group; CoP, Ceramic-on-polyethylene control group.

The results of blood metal ion analysis are shown in Table 3 and Figure 1. At a mean follow-up of 15 years, elevated blood metal ion concentrations of cobalt, chromium and titanium were measured in the metal-on-metal study group, with a statistically significant difference compared to the control groups (Figure 1).

### 3.4. Phenotypic Analysis of Peripheral Blood Mononuclear Cells

**Figure 2:**
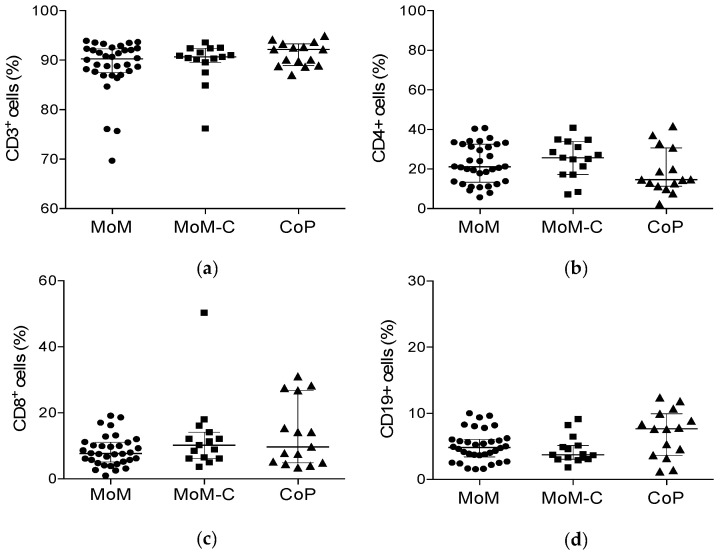
Scatter plots summarizing the results of phenotypic analysis of lymphocyte subpopulations as measured by flow cytometry. The figure presents the percentages as frequencies of CD3^+^ cells of: (**a**) CD3^+^ T cells; (**b**) CD4^+^ T helper cells (**c**) CD8^+^ cytotoxic T cells; and (**d**) CD19^+^ B cells. No statistically significant differences were found between the groups. Vertical scatterplot lines show medians with interquartile range. MoM, metal-on-metal study group; MoM-C, metal-on-metal control group; CoP, ceramic-on-polyethylene group.

**Figure 3:**
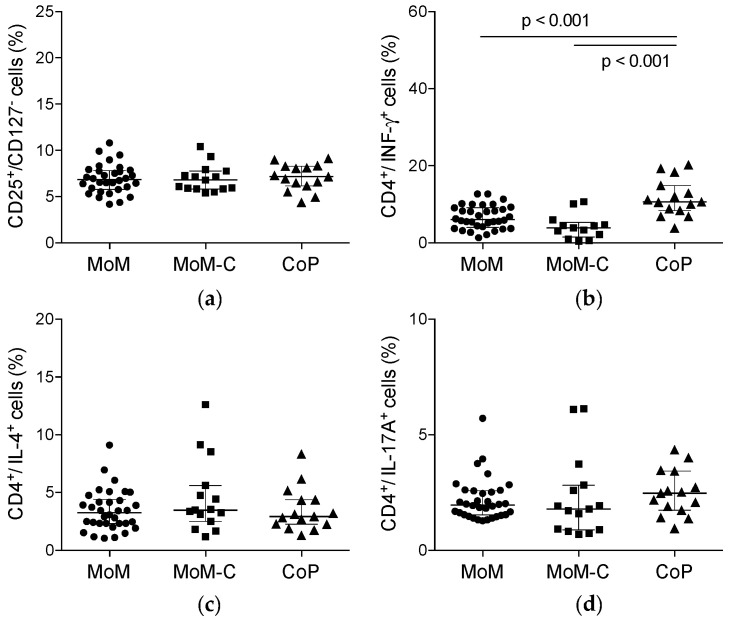
Scatter plots summarizing the results of phenotypic analysis of lymphocyte subpopulations as measured by flow cytometry. The figure presents the percentages as frequencies of CD3^+^ cells of: (**a**) CD25^+^/CD127^−^ regulatory T cells as frequencies of mononuclear cells; (**b**) CD4^+^/IFN-γ^+^ Type 1 (Th1) T helper cells; (**c**) CD4^+^/IL-4^+^ Type 2 (Th2) T helper cells; and (**d**) CD4^+^/IL-17A^+^ Th17 T helper cells. Lines with *p* values connect groups with significant differences. Vertical scatterplot lines show medians with interquartile ranges. MoM, metal-on-metal study group; MoM-C, metal-on-metal control group; CoP, ceramic-on-polyethylene group.

The results of cytometric analysis of lymphocyte subpopulations are presented in Table 4, Figure 2 and Figure 3. In summary, we found no statistically significant differences regarding the analyzed lymphocyte subsets between patients with elevated blood metal ion levels and the control groups except for the lymphocyte counts of IFN-γ producing Th1 helper cells. The mean percentages of IFN-γ^+^ T helper cells were significantly higher in patients with ceramic-on-polyethylene THA compared to patients with metal-on-metal THA, regardless of the measured blood metal ion levels (Table 4 and Figure 2).

In the metal-on-metal study group, bivariate analysis using Spearman correlation coefficient revealed a negative correlation between chromium ion concentrations and IL-4^+^ Th2 cells (ρ= −0.565, *p* = 0.001) and between titanium ion levels and CD3^+^ lymphocytes (ρ= −0.403, *p* = 0.018). However, multivariate linear regression analysis using age, gender, cobalt, chromium and titanium as independent variables did not confirm these correlations for CD3^+^ cells or Th2 cells.

### 3.5. Comparison of Lymphocyte Cell Counts between Patients with ALTR and Patients without ALTR

We investigated if the presence of periprosthetic adverse local tissue reactions secondary to metal wear was associated with changes in peripheral blood mononuclear cell subpopulations and compared the lymphocyte cell counts between patients with ALTR (*n* = 11) and patients without ALTR (*n* = 16). With respect to demographic parameters, both groups were comparable: mean patient age was 68 years (SD 5.4) and 66 years (SD 8.7, *p* = 0.446); mean follow-up duration was 15.5 years (SD 1.8) and 15.7 years (SD 1.2, *p* = 0.520); and mean BMI was 26 kg/m^2^ (SD 3.1) and 27 kg/m^2^ (SD 5.4, *p* = 0.484) in the ALTR group compared to the non-ALTR group, respectively. Interestingly, no differences in mean HHS was seen between the two groups (89 ± 10.1 points vs. 89 ± 15.8 points, *p* = 0.486). In the ALTR group, significantly higher cobalt ion concentrations were measured compared to patients without ALTR (*p* = 0.0003, Table 5). Comparison of lymphocyte cell counts showed no statistically significant differences between both groups (Table 5).

## 4. Discussion

Every metal implant releases metal ions in a biological environment through different modes of corrosion. In total hip arthroplasty, it has been shown that the accumulation of metal ions due to fretting corrosion at modular taper junctions can cause adverse effects such as implant loosening and the development of adverse local tissue reactions (ALTR). High systemic blood metal ion levels have been measured in patients with MoM THA due to excessive wear and previous studies could demonstrate a suppressive effect of elevated blood metal ion concentrations on circulating T lymphocytes in the short term [16,17,18,19,20]. The aim of the present study was to investigate peripheral lymphocyte subpopulations in patients with elevated blood metal ion concentrations after small diameter MoM THA at long-term follow-up. Our hypothesis was that increased blood metal ion levels would be associated with changes in the levels of peripheral lymphocyte subpopulations. The second purpose of this study was to compare levels of lymphocyte subpopulations between patients with and without ALTR at long-term follow-up. We hypothesized that the presence of an implant-related T cell-mediated immunological response, suspected in patients with ALTR, would influence the levels of systemic lymphocyte subsets.

The results of the present study demonstrated lower percentages of IFN-γ^+^ type 1 T helper cells (Th1) in patients with MoM THA as compared to patients with ceramic-on-polyethylene THA. With regard to the other analyzed lymphocyte subtypes, no statistically significant differences between the three groups were found, and no differences in the levels of lymphocyte subpopulations were seen between patients with and without ALTR in this cohort.

Hart et al. investigated lymphocyte subpopulations in the peripheral blood of patients with MoM hip resurfacings and found a statistically significant decrease in the levels of CD8^+^ cytotoxic T cells compared to patients with standard metal-on-polyethylene THA [18]. The authors reported a threshold ion level of 5 ng/mL of combined cobalt and chromium concentrations to be associated with reduced CD8^+^ T-cell counts. Another study by Granchi et al. reported reduced levels of CD4^+^ T helper cells and CD8^+^ cytotoxic T cells in patients with loosened hip replacements made of co–chromium alloys [19]. In contrast to these findings, there was no evidence of a suppressive effect of a long-term metal ion exposure on CD4^+^ and CD8^+^ T cell counts after 15 years in our cohort. Catelas et al. investigated lymphocyte subpopulations in patients with failed MoM hip implants [16]. They found overall lower percentages of memory T cells and IFN-γ^+^ Th1 cells in patients with failed MoM hip replacements who demonstrated ALTR compared to patients without ALTR [16]. The authors discussed either a toxic effect of metal wear products or a sequestration effect of T lymphocytes at the implant site due to a cell-mediated hypersensitivity reaction accountable for the decrease in systemic T cell counts [16,19]. They concluded that a sequestration effect at the implant site rather than a toxic effect of elevated metal ion concentrations was responsible for the decrease in Th1 cells, because other lymphocyte subgroups, such as B cells and NK cells, did not show differences in their blood concentrations [16]. In accordance to these study results, we found decreased levels of Th1 cells in patients with MoM bearings in comparison to patients following ceramic-on-polyethylene THA. Bivariate correlation analysis revealed a correlation between blood metal ion concentrations and the levels of IL-4^+^ Th2 and IL-17A^+^ T helper cells, but no correlation was evident between Th1 cell counts and blood metal ion levels, and no differences in the levels of lymphocyte subpopulations were seen between patients with and without ALTR in this cohort.

While the non-specific immune response to wear particles in terms of a macrophage-mediated foreign body reaction is well studied, the role of the adaptive immune system in the development of metal wear related complications and the immunological pathways that lead to ALTR formation and implant loosening are still not completely understood. Metal ions are able to bind to serum proteins and form hapten-like metal–protein complexes, which can activate the immune system as potential antigens and might cause a delayed type IV hypersensitivity reaction. Th1 helper cells play an important role in this cell-mediated immune response. When activated, Th1 cells release different cytokines such as IFN-γ and IL-2, which recruit macrophages and induce a proinflammatory cascade [27]. Histopathological analysis of pseudotumor-like tissues around failed MoM hip replacements demonstrated typical histological patterns with extensive connective tissue necrosis, macrophage infiltrates and marked lymphocyte infiltration [10,28,29]. In some cases, ALTR occur in the absence of excessive metal wear. These tissue reactions typically demonstrate high ALVAL-scores with diffuse and perivascular T and B cell infiltrates, which are suggestive for a lymphocyte-dominated type IV hypersensitivity reaction [28,29]. Hallab et al. demonstrated that metal ions are able to induce the production of IFN-γ in T lymphocytes of patients following THA in an in-vitro study and the authors suggested that a Th1 type lymphocyte response was likely associated with metal induced reactivity [13]. These findings support the hypothesis that the development of ALTR in patients with MoM hip replacements could be partly immunologically mediated as a result of T cell activation [30]. Hailer et al. reported an association between metal ion levels and the presence of ALTR in patients with MoM hip resurfacings and found a correlation of increased cobalt ion concentrations with the level of activated HLA-DR^+^ T helper cells [30]. In accordance to these findings, we found higher cobalt ion levels in patients with ALTR compared to patients without ALTR in the present study [31,32,33]. However, the results of this study did not demonstrate any differences in T cell counts between patients with ALTR and patients without ALTR. In contrast to the before mentioned studies which investigated lymphocyte subpopulations in patients with failed total hip replacements, most of the patients in our cohort were asymptomatic with a well-functioning THA. In addition, metal wear particles and corrosion products may differ in size and biological reactivity between patients with small head MoM THA and patients with MoM hip resurfacings, which might influence the immunological response and limits the comparison between the studies.

There are limitations to this study that have to be acknowledged. Due to the retrospective study design blood metal ion analysis and flow cytometry analysis were performed at a single time point after a mean follow-up of 15 years and no sequential analysis was performed for each patient in the study group. However, longitudinal studies showed that blood metal ion levels in patients with well-functioning small head metal-on-metal bearings did not tend to increase over time at long-term follow-up [34,35,36]. On the other hand, we consider the long-term follow-up duration of 15 years to be a strength of this study. In addition, the demographic parameters of the study and the control group were comparable and the high percentage of asymptomatic patients in the MoM group makes this study cohort representative for other populations with well-functioning THA, because age as well as pain might influence absolute T cell counts [18,37]. However, the difference in the follow-up duration between the metal-on-metal study group and the ceramic-on-polyethylene control group has to be discussed as a limitation of this study, which could have resulted in a potential selection bias. In the ceramic-on-polyethylene control group, patients were recruited based on their age at time of follow-up, because we intended to avoid larger differences in demographic variables such as gender or patient age, as both factors affect the peripheral expression of lymphocytes in healthy individuals [37]. Although the number of patients included in the present study is comparable to those of other studies that have reported significant differences in lymphocyte subpopulations in patients with MoM hip implants, the relatively low number of patients has to be considered as a limitation and a larger sample size could have increased the statistical power of the study.

In conclusion, no adverse systemic effects at long-term follow-up with respect to peripheral blood lymphocyte subpopulations in patients following total hip replacement with a small head MoM articulation could be detected in the present study. We found a significant decrease in the levels of IFN-γ^+^ Th1 cells in patients with MoM THA compared to the control group and bivariate correlation analysis revealed a correlation between blood metal ion concentrations and the levels of IL-4^+^ Th2 and IL-17A^+^ T helper cells in this cohort. Although MoM bearings are rarely used in THA nowadays, the potential adverse local effects of accumulating metal ions in the periprosthetic environment will continue to be of clinical relevance because metal ions are continuously released by every metal implant due to wear and corrosion, especially in modular joint replacements. The interactions between metal ions and the immune system as well as their influence on osteoclastogenesis and bone metabolism are still not completely understood and the pathomechanisms of these interactions with respect to implant failure and the development of adverse local soft tissue reactions should be further investigated.

## Figures and Tables

**Table 1:** Overview of the leucocyte subgroups analyzed in the present study.

Leucocytes	Marker	Main Functions
T cells	CD3^+^	
-T helper cells	CD4^+^	Mediators of the adaptive immune system; release cytokines that influence/activate other lymphocytes
-T_h_1 helper cells	IFN-γ	Produce IFN-γ, which leads to a cell-mediated immune response; key role in delayed type IV hypersensitivity reactions
-T_h_2 helper cells	IL-4	Produce IL-4 among others, which leads to a humoral immune response, typically against extracellular bacteria and parasites;
-T_h_17 helper cells	IL-17A	Produce IL-17, a pro-inflammatory cytokine linked to autoimmune related diseases such as rheumatoid arthritis
-T regulatory cells	CD25^+^/CD127^−^	Modulators of the immune system; inhibit proliferation of effector T cells; maintain immunological tolerance to self-antigens
-Cytotoxic T cells	CD8^+^	Effector cells that induce apoptosis in virus-infected cells or tumor cells by secretion of different cytotoxins
B cells	CD19^+^	Major cells involved in the creation of antibodies (immunoglobulin), known as the humoral immunity of the adaptive immune system;
NK (Natural killer) cells	CD16^+^/CD56^+^	Part of the innate immune system; function similar to that of cytotoxic T cells; destroy virus-infected cells and tumor cells
Monocytes/Macrophages	CD14^+^	Phagocytic leukocytes; differentiate into tissue resident macrophages; activate T lymphocytes by antigen presentation

**Table 2:** Demographic data of the metal-on-metal study group (MoM, *n* = 34), the metal-on-metal control group (MoM-C, *n* = 15) and the ceramic-on-polyethylene group (CoP, *n* = 15).

Parameter	MoM Group (*n* = 34)	MoM-C Group (*n* = 15)	CoP Group (*n* = 15)	*p*-Value MoM vs. MoM-C	*p*-Value MoM vs. CoP	*p*-Value MoM-C vs. CoP
Age at follow-up ^†^ (years)	67.8 (50–78)	69.1 (56–81)	61.1 (27–82)	1.000	0.446	0.387
Female gender (%)	47	27	53	0.221	0.762	0.264
Body mass index ^†^ (kg/m^2^)	27 (18–40)	29 (21–41)	28 (20–35)	0.347	0.774	0.815
Time of follow-up ^†^ (years)	15.6 (13.7–18.3)	14.4 (13.3–16.9)	1.5 (0.3–5.9)	0.080	<0.001 *	0.002 *
Pat. with bilateral THA (%)	70	13	13	<0.001 *	<0.001 *	1.000
Harris Hip Score ^†^ (points)	90 (50–100)	88 (61–100)	-	0.641	-	-
Metal ion level (Co + Cr) ^†^ (µg/L)	6.88 (2.04–41.50)	0.78 (0.32–0.97)	0.83 (0.29–1.07)	<0.001 *	<0.001 *	1.000

^†^ The values are given as the mean, with the range in parentheses; * indicates statistically significant differences between the groups.

**Table 3:** Results of blood metal ion analysis (the values are given in µg/L).

Parameter		Metal-on-Metal Study Group (*n* = 34)	Metal-on-Metal Control Group (*n* = 15)	Ceramic-on-Polyethylene Group (*n* = 9)
Cobalt	Mean (SD)	2.87 (4.62)	0.41 (0.12)	0.20 (0.16)
	Median (range)	1.53 (0.42–24.75)	0.42 (0.24–0.63)	0.16 (0.08–0.61)
Chromium	Mean (SD)	4.01 (4.91)	0.37 (0.15)	0.64 (0.22)
	Median (range)	2.10 (0.66–22.69)	0.34 (0.06–0.64)	0.65 (0.21–0.93)
Titanium	Mean (SD)	4.94 (6.24)	2.09 (1.03)	1.73 (1.23)
	Median (range)	2.96 (0.99–27.25)	1.94 (0.44–3.83)	1.56 (0.47–3.92)

Note: Statistically significant differences between the three groups are displayed in Figure 1.

**Table 4:** Summary of leucocyte cell counts of the study and the control groups (the values are given in percentages, as frequency of parents).

Leucocytes	Metal-on-Metal Study Group (*n* = 34)	Metal-on-Metal Control Group (*n* = 15)	Ceramic-on-Polyethylene Group (*n* = 15)	*p*-Value MoM vs. MoM-C	*p*-Value MoM vs. CoP	*p*-Value MoM-C vs. CoP
CD3^+^	88.85 (5.40)	89.61 (4.28)	91.27 (2.37)	1.000	0.351	1.000
CD4^+^	22.70 (9.92)	25.19 (9.63)	18.76 (11.48)	0.713	0.435	0.206
CD8^+^	8.51 (4.58)	12.60 (11.22)	13.60 (10.01)	0.520	0.613	1.000
CD19^+^	5.04 (2.36)	4.47 (2.04)	6.97 (3.58)	1.000	0.340	0.113
CD14^+^	12.17 (5.18)	11.64 (4.95)	9.71 (2.89)	0.930	0.217	0.501
CD16^+^/CD56^+^	6.90 (4.49)	8.80 (5.41)	6.85 (4.11)	0.390	1.000	0.488
CD25^+^/CD127^−^	6.91 (1.56)	6.94 (1.45)	7.09 (1.43)	1.000	1.000	1.000
IFN-γ	6.68 (3.03)	4.21 (3.23)	11.65 (4.79)	0.092	<0.001 *	<0.001 *
IL-4	3.46 (1.77)	4.60 (3.18)	3.51 (1.89)	0.847	1.000	0.973
IL-17	2.19 (0.92)	2.29 (1.77)	2.48 (0.99)	1.000	0.871	0.469

The values are given as the mean with the standard deviation in parentheses. IFN-γ, interferon-gamma; IL, interleukin. * indicates statistically significant differences between the groups.

**Table 5:** Comparison of leucocyte cell counts and metal ion levels between patients without ALTR and patients with ALTR (the values are given in percentages, as frequency of parents).

Leucocytes/Blood Metal Ion Levels	Patients without ALTR (*n* = 16)	Patients with ALTR (*n* = 11)	*p*-Value
CD3^+^	86.95 (7.05)	90.78 (2.81)	0.101
CD4^+^	23.71 (8.85)	23.25 (11.99)	0.910
CD8^+^	8.92 (4.94)	9.40 (4.86)	0.802
CD14^+^	12.82 (6.33)	9.84 (4.20)	0.184
CD19^+^	5.27 (2.33)	4.65 (2.15)	0.487
CD16^+^/CD56^+^	5.99 (4.28)	6.24 (3.70)	0.877
CD25^+^/CD127^−^	6.88 (1.38)	6.21 (2.73)	0.409
IFN-γ	6.04 (2.93)	6.94 (2.84)	0.439
IL-4	3.15 (1.74)	4.06 (1.92)	0.210
IL-17	2.44 (1.19)	2.00 (0.59)	0.273
Cobalt ion level (µg/L)	1.06 (0.47)	3.63 (3.85)	<0.001 *
Chromium ion level (µg/L)	4.38 (5.67)	2.62 (2.22)	0.178
Titanium ion level (µg/L)	4.40 (4.45)	7.44 (9.27)	0.865

The values are given as the mean with the standard deviation in parentheses; * indicates statistically significant differences between the two groups; IFN-γ, interferon-gamma; IL, interleukin.
